# IL-6 597A/G (rs1800797) and 174G/C (rs1800795) Gene Polymorphisms in the Development of Cervical Cancer in Lithuanian Women

**DOI:** 10.3390/medicina57101025

**Published:** 2021-09-26

**Authors:** Agne Vitkauskaite, Joana Celiesiute, Vijoleta Juseviciute, Kristina Jariene, Erika Skrodeniene, Gabriele Samuolyte, Ruta Jolanta Nadisauskiene, Daiva Vaitkiene

**Affiliations:** 1 Department of Obstetrics and Gynaecology, Medicine Academy, Lithuanian University of Health Sciences, 44307 Kaunas, Lithuania; joana.celiesiute@lsmu.lt (J.C.); kristina.jariene@lsmu.lt (K.J.); ruta.nadisauskiene@lsmuni.lt (R.J.N.); daiva.vaitkiene@lsmuni.lt (D.V.); 2 Department of Laboratory Medicine, Lithuanian University of Health Sciences Hospital, 50161 Kaunas, Lithuania; vijoleta.juseviciute@kaunoklinikos.lt; 3 Department of Laboratory Medicine, Medicine Academy, Lithuanian University of Health Sciences, 44307 Kaunas, Lithuania; erika.skrodeniene@lsmuni.lt; 4 Medicine Academy, Lithuanian University of Health Sciences, 44307 Kaunas, Lithuania; gabriele.samuolyte@stud.lsmu.lt

**Keywords:** cervical cancer, gene polymorphisms, HPV

## Abstract

Our study aimed to evaluate the distribution of genotypes and allele frequencies of IL-6 597A/G (rs1800797) and 174G/C (rs1800795) polymorphisms in HPV infected and uninfected healthy women and cervical cancer patients. A PCR based Multiplex HPV genotyping test kit was used for in vitro detection and differentiation of high risk HPV genotypes. Genotyping of two polymorphisms, IL-6 597A/G (rs1800797) and 174G/C (rs1800795), was performed using the KASP genotyping assay kit. Cervical cancer patients were more likely to be HPV positive than control patients. Allele C of IL-6 rs1800795 was associated with a higher risk of cervical cancer by 2.26-fold and genotype CC by 5.37-fold. Genotype CC of IL-6 rs1800795 was more frequent in the HPV positive group compared with the HPV negative group (*p* = 0.002). Allele G of IL-6 rs1800797 was more frequently found in women with HPV16/HPV18 compared to other HPV types (*p* = 0.045). Women with AA genotypes of IL-6 rs1800797 were less frequently infected with HPV16/HPV18 compared to other HPV types (*p* = 0.045). The major finding of the study is the significant association of C allele and CC genotype of IL-6 1800795 gene with cervical cancer in the Lithuanian population. Genotype CC of IL-6 rs1800795 has a significant association with HPV infection as well.

## 1. Introduction

Cervical cancer remains a major health problem worldwide, especially in Lithuania, where morbidity and mortality rates are one of the highest among all Baltic countries [1]. Although the participation of women in the screening program is not sufficient, this alone cannot explain the failures of the screening program. Therefore, the search of risk factors and creation of prevention guidelines is of utmost importance. It is proven that cervical intracellular squamous epithelium abnormalities and cancer are closely related to persisting human papillomavirus (HPV) infection. The persistence of infections by high-risk HPV types is the single most significant risk factor for malignant progression [2]. This virus infects the epithelium exclusively, replicating only in fully matured epithelium cells, affects standard cell cycle control, and promotes an uncontrollable cell division, causing genetic damage [3]. HPV infection alone is not enough for complicated processes of cellular change, and it is not always possible to make the association between HPV infection and cervical intracellular squamous epithelium abnormalities [4]. Clinical data show that the development of cervical cancer is the multifactorial process in which not only HPV takes place, but also other risk factors such as smoking, family history, age, or immunosuppression [5,6]. In our previous study, we have found increased serum levels of inflammatory markers—TNF-α, IFN-β, IL-1β, sTREM-1, IL-6—in cervical cancer patients. The changes of cytokines are most likely cervical cancer-related and support the hypothesis of systemic inflammation in cervical cancer [7].

Worldwide, a lot of attention goes into the search for immune response and genetic factors that might predispose to cervical cancer. Gene mutations, especially polymorphisms in the promoter region, can affect gene transcription, resulting in abnormal expression of the corresponding mRNAs and dysfunction of the expressed proteins. These mutations can also influence the susceptibility of individuals to cancer [8,9].

Scientists cannot find one separate gene mutation, which would be responsible for cervical cancer development. Polymorphisms in genes related to immunity have been reported to influence several diseases, including viral infection [10]. That is why there are increasingly more studies searching for candidate gene variants (single nucleotide polymorphisms (SNP) included) related to increased risk of cervical cancer. Detection of such genes could lead to screening of women at risk or to specific treatment of patients in the future. Several genes and their polymorphisms associated with the risk of development of cervical cancer have been detected [11,12]. However, many questions about gene polymorphism and disease are unanswered. Moreover, the results vary in different populations. The potential confounding factors, such as race, age, and geographical distribution, may exist. Interleukin 6 (IL-6) is one of the most widely recognized cytokines. It can regulate immune responses and cell proliferation and differentiation [13]. Peng meta-analysis results show that seventy-eight studies reported the association between polymorphism rs1800795 and cancer risk. Seventeen studies reported the association between polymorphism rs1800797 and cancer risk, and twelve studies reported the association between IL-6 promoter polymorphisms and cancer prognosis [14].

Our study’s aim was to evaluate the distribution of genotypes and allele frequencies of polymorphisms IL-6 597A/G (rs1800797) and 174G/C (rs1800795) among healthy women and cervical cancer patients, looking for a link between gene polymorphism and cervical cancer in the Lithuanian population.

## 2. Materials and Methods

### 2.1. Patients and Study Design

The study was carried out on 92 patients with histology proven cervical cancer, and the control group consisted of 84 healthy women with the cervicovaginal cytology test negative for intraepithelial lesions or malignancy (NILM) confirmed by liquid based cytology test (SurePath, Becton Dickinson, Burlington, VA, USA) and reported according to the 2014 Bethesda System formed the control group. All women were treated in the Department of Obstetrics and Gynaecology of Hospital of Lithuanian University of Health Sciences Kauno klinikos.

The exclusion criteria were for all study participations as follows: women with any autoimmune diseases, active or chronic infections, cardiovascular diseases, connective tissue diseases, a history of malignant tumors and younger than 18 years, diabetes, and pregnancy. None of the patients previously received immunosuppressive treatment, radiotherapy, or chemotherapy or had previously received treatment for *carcinoma* in situ or HSIL.

The study was approved by the Kaunas Regional Biomedical Research Ethics Committee (No. BE-2-80/2018), and written consent from all subjects was received.

### 2.2. HPV Detection and Genotyping

Liquid based cervical samples were obtained for genomic DNA isolation for HPV determination. A Polymerase Chain Reaction (PCR) based Multiplex HPV genotyping test kit (DiaMex, New York, NY, USA) was used for in vitro detection and differentiation of HPV 24 genotypes (HPV types 16, 18, 26, 31, 33, 35, 39, 45, 51, 52, 53, 56, 58, 59, 66, 68, 73, and 82) in cervical samples.

### 2.3. DNA Extraction and Genotyping

Genomic DNA was extracted from the blood using QIAamp DNA Blood Mini Kit (Qiagen, Venlo, The Netherlands). The concentration and purity of DNA were evaluated using a spectrophotometer Epoch (Biotek Instruments, Winooski, VT, USA). All the measurements were performed according to the manufacturer’s manual.

Genotyping of two polymorphisms IL-6 597A/G (rs1800797) and 174G/C (rs1800795), was performed using the KASP genotyping assay kit (LGC Biosearch Technologies, Hoddesdon, UK) on Real-Time PCR system RotorGene Q (Qiagen). The KASP (Competitive Allele Specific PCR) genotyping system was chosen as a reliable and high accurate bi-allelic scoring method. KASP assay mix is designed to work with specific polymorphism to be targeted and consists of two competitive, allele-specific forward primers and one standard reverse primer. One allele specific primer is labeled with FAM dye and the other with HEX dye. During thermal cycling, the allele-specific primer binds to the template and generates the signal. If the genotype of relevant polymorphism was homozygous, only one fluorescent signal was generated. If the genotype was heterozygous, the mix of both FAM and HEX signals was generated. Genotypes were clustering using scatter plot analysis software.

### 2.4. Statistical Analysis

Statistical data analysis was performed using the statistical package IBM SPSS 23.0. The Kolmogorov–Smirnov test was employed to determine distribution of quantitative data. For the analysis of normally distributed data, the Student t test (for comparison of the mean of two samples) was applied. The Chi-square test was used to determine whether a relationship exists between qualitative data.

Differences comparing the groups were considered statistically significant when a *p* value was less than 0.05. The study power analysis was done and determined as 0.8. Analyzing the impact of a factor on disease development, the odds ratio (OR) was calculated. The factor was considered significant if the lower and upper limits of the OR 95% confidence interval (CI) were less than or greater than 1.

## 3. Results

A total of 175 women aged 22–85 years were included in the analysis. The mean age of the cervical cancer patients and NILM groups was significantly different (*p* < 0.001), but we used random function to get an equal control group. Table 1 shows the characteristics of the study population; 90 women in the cervical cancer group were HPV positive, and this percentage significantly differed from the percentage of HPV-positive women in the control group (98.9% vs. 14.3%, *p* < 0.001).

Final number of reach group differs due to missing data in 1–17 cases. χ^2^ chi-square tests were used to determine whether a relationship exists between qualitative data.

The frequencies of alleles and genotypes of IL-6 rs1800797 and IL-6 rs1800795 in the control and cervical cancer group are presented in Table 2. IL-6 rs1800797 genotypes and allele frequencies were not statistically different between women with cervical cancer and healthy controls. Allele C of IL-6 rs1800795 and genotype CC of IL-6 rs1800795 were more frequent among women with cervical cancer than in controls (74.2% vs. 56%, *p* < 0.012 and 29.2% vs. 7.1%, *p* < 0.001 respectively). Allele G of IL-6 rs1800795 was less frequent in cervical cancer group compared with controls (70.8% vs. 92.9%, *p* < 0.001). Furthermore, genotype GG of IL-6 rs1800795 was more frequent among control women than in the cervical cancer group (44% vs. 25.8%, <0.012). Allele C of IL-6 rs1800795 was associated with higher risk by 2.26-fold (95% CI 1.19–4.29), and genotype CC was associated with higher risk by 5.37-fold (95% CI 2.08–13.84) of cervical cancer. Allele G of IL-6 rs1800795 was associated with lower risk by 5.3-fold (95% CI 0.07–0.48), and genotype GG was associated with lower risk by 2.3-fold (95% CI 0.23–0.84) of cervical cancer (Table 2).

IL-6 rs1800797 and IL-6 rs1800795 genotypes and allele frequencies between HPV infected and uninfected healthy controls and women with cervical cancer are shown in Table 3. Allele G of IL-6 rs1800795 was less frequent, but genotype CC was more frequent in HPV positive group compare with HPV negative (73.5% vs. 91.8%, respectively, *p* = 0.002) and (26.5%, vs. 8.2%, respectively, *p* = 0.002). There was a tendency for higher distribution of C allele of IL-6 rs1800795 in HPV positive women when compared to negative (71.4% and 57.5%, respectively, *p* = 0.059). The frequency of cases with GG genotype of IL-6 rs1800795 in HPV positive women was lower in comparison to HPV negative women (28.6% vs. 42.5%, respectively, *p* = 0.059)*,* but did not show a significant association. Allele G of IL-6 rs1800795 was associated with lower risk by 4-fold (95% CI 0.1–0.64) of HPV infection. However, genotype CC of IL-6 rs1800795 was associated with a higher risk by 4-fold (95% CI 1.56–10.91) of HPV infection.

IL-6 rs1800797 and IL-6 rs1800795 gene polymorphisms and HPV genotype distribution in the Lithuanian population are presented in Table 4. Allele G of IL-6 rs1800797 was more frequent find in women with HPV16 and HPV18 compared to women with other HPV type (75.0%, n = 54 and 53.8%, n = 14 respectively, *p* = 0.045). Women with AA genotypes of IL-6 rs1800797 were less frequent infected with HPV16 and HPV18 compared to other HPV types (25.0%, n = 18 and 46.2%, n = 12 respectively, *p* = 0.045). We found no association of IL-6 rs1800795 genotypes and allele frequencies between HPV16, HPV18, and other HPV types.

The genotype and allele frequencies of IL-6 rs1800797 and IL-6 rs1800795 gene polymorphism were not associated with tumor progression (Table 5).

## 4. Discussion

The aim of our study was to evaluate the potential contribution of specific IL-6rs1800797 and IL-6rs1800795 polymorphisms to the susceptibility for cervical cancer. Our data show that IL-6 rs1800797 polymorphism is not a risk factor for cervical cancer in Lithuania. However, we observed that the HPV16 and HPV18 types, the most prevalent and aggressive types worldwide, are predominant in cases with G allele of IL-6 rs1800797. Our study results demonstrated an increased OR for C allele and CC genotype of IL-6 rs 1800795 gene in cervical cancer cases compared to controls. Allele G and genotype GG of IL-6 rs1800795 were associated with a lower risk of cervical cancer, but allele C and genotype CC were associated with a higher cervical cancer risk in the Lithuanian population. Other studies confirmed this association in various populations and suggested that these genetic alterations were also linked to cervical cancer. A meta-analysis by Liu and colleagues, including 1210 cervical cancer cases and 1525 controls from 5 studies from Brazil, China, India, Austria, and Finland, demonstrated that rs1800795 polymorphisms of IL-6 gene (G vs. C alleles) might be associated with susceptibility to cervical cancer. C genotype of IL-6rs1800795 was associated with higher cervical cancer risk [15]. Duan and colleague’s results indicate that the C genotype of IL-6-174G>C polymorphism might be associated with higher cervical cancer risk as well [16]. The frequencies of C allele of IL-6 rs1800795 were significantly higher in cervical cancer cases, and logistic regression analysis indicated that cervical cancer risks were significantly higher in C allele carriers than those with GG genotype (GC+CC versus GG) in Chinese women [17]. Other studies confirmed that allele G and genotype GG of IL-6 rs1800795 might have a protective effect against cervical cancer development [18].

Zidi‘s study in Tunisian population data demonstrated different results. The tested IL-6 six SNPs’ genotype distribution, including IL-6 rs1800797 and IL-6rs1800795, was similar between both cervical cancer cases and control groups. They concluded that more studies are required for confirming or ruling out the association of these variants with cervical cancer [18]. Different results compared to our data were observed in Shi’s study, where IL-6 rs1800797 polymorphism confers a higher risk of cervical cancer in the Chinese population [19]. Gupta and colleagues found that the presence of −597 G allele of IL-6-597A/G increases the risk of cervical cancer in the study population by up to 6.2 times (*p* < 0.001) [12]. The limited number of studies and sample size for such polymorphism may reduce the results’ reliability and affect the assessment of associations between these polymorphisms and cervical cancer susceptibility. The ethnically distinct populations could be a possible explanation for the opposed results of the separate studies. In contrast to Gupta et al. and Shi et al., our study did not detect any statistically significant association between IL-6 rs1800797 polymorphisms and susceptibility to cervical cancer.

Our data show that genotype CC of IL-6 rs1800795 was more frequent in HPV positive women than HPV negative women. These observations suggest that IL-6rs1800795 and high-risk HPV infection may be cooperatively associated with cervical carcinogenesis. However, we found no association of IL-6 rs1800795 genotypes and allele frequencies between HPV16, HPV18, and other HPV types. Junior’s study data shows any significant association between the IL-6+174G>C polymorphism and HPV infected women or uninfected controls or between HSIL and LSIL subgroups. However, taking into account the fact that individuals from the control group could have been previously infected by HPV that was spontaneously eliminated, and based on findings, Junior and colleagues hypothesized that the IL-8 + 396 G allele and GG genotypes could play a role in the risk of HPV infection in a Brazilian study population [20].

We did not find an association between the genotype and allele frequencies of IL-6 rs1800797 and IL-6 rs1800795 gene polymorphism and tumor progression in cervical cancer cases. It is difficult to find other studies which confirm such associations. However, Peng data show that rs1800795 and rs1800796 were associated with a significantly higher risk of cancer in Asia and Caucasians, rs1800797 was associated with a significant risk of cancer in Caucasian, but not in Asia. Furthermore, IL-6 promoter polymorphisms were significantly associated with the prognosis of cancer. Considering these promising results, IL-6 promoters, including rs1800795 and rs1800797, may be a tumor marker for cancer therapy [14].

The interactions between genetic and environmental factors and cervical cancer development were not evaluated in our analysis.

## 5. Conclusions

The major finding of our study is the significant association of C allele and CC genotype of IL-6 1800795 gene with cervical cancer in the Lithuanian population. Genotype CC of IL-6 rs1800795 has a significant association with HPV infection as well. In our study, the genotype and allele frequencies of IL-6 rs1800797 and IL-6 rs1800795 gene polymorphisms were not associated with tumor stage.

## Figures and Tables

**Table 1:** Characteristics of the study population.

Characteristic	Cervical Cancer, *N* = 92 n (%)	Control, *N* = 84 n (%)	χ^2^	*p* Value
Age (mean (SD))	54.84 (15.64)	48.45 (9.35)	–	<0.001
Married status Yes No	57 (71.3) 25 (28.7)	56 (69.1) 25 (30.9)	0.09	0.769
Sexual partner 1 >1	25 (33.3) 50 (66.7)	28 (37.8) 46 (62.2)	0.33	0.566
Oral contraceptive users Yes No	3 (3.8) 77 (96.3)	15 (19.2) 63 (80.8)	9.38	0.002
Smokers Current or past Never	31 (37.8) 51 (62.2)	11 (14.1) 67 (85.9)	11.60	0.001
Figo staging Stage I Stage II Stage III Stage IV	6 (7.1) 37 (43.5) 31 (36.5) 11 (12.9)	– – – –	– – – –	
HPV status Positive Negative	90 (98.9) 1 (1.1)	12 (14.3) 72 (85.7)	128.63	<0.001

**Table 2:** The allele and genotype frequency distributions of IL-6 both polymorphisms rs1800797, IL-6 rs1800795 in cervical cancer patients and controls.

	Study Population		χ^2^	*p* Value	OR (95% CI)
	Cervical Cancer, *N* = 89 n (%)	Control, *N* = 84 n (%)			
IL-6 rs1800797 Allele A G Genotype AA AG GG	66 (74.2) 64 (71.9) 25 (28.1) 41 (46.1) 23 (25.8)	71 (84.5) 57 (67.9) 27 (32.1) 44 (52.4) 13 (15.5)	2.82 0.34 0.34 0.69 2.82	0.093 0.561 0.561 0.406 0.093	0.53 (0.25–1.21) 1.21 (0.63–2.32) 0.83 (0.43–1.58) 0.78 (0.43–1.41) 1.90 (0.89–4.06)
IL-6 rs1800795 Allele C G Genotype CC CG GG	66 (74.2) 63 (70.8) 26 (29.2) 40 (44.9) 23 (25.8)	47 (56.0) 78 (92.9) 6 (7.1) 41 (48.8) 37 (44.0)	6.32 13.96 13.96 0.26 6.32	0.012 <0.001 <0.001 0.611 0.012	2.26 (1.19–4.29) 0.19 (0.07–0.48) 5.37 (2.08–13.84) 0.86 (0.47–1.56) 0.44 (0.23–0.84)

χ^2^, chi-square test were used to determine whether a relationship exists between qualitative data. OR, odds ratio; CI, confidence interval.

**Table 3:** Genotype and allele frequencies of IL-6 polymorphisms rs1800797, IL-6 rs1800795 and association with HPV in cervical cancer patients and controls.

	HPV		χ^2^	*p* Value	OR (95% CI)
	Positive *N* = 102 n (%)	Negative *N* = 73 n (%)			
IL-6 rs1800797 Allele A G Genotype AA AG GG	75 (76.5) 68 (69.4) 30 (30.6) 45 (54.9) 23 (23.5)	61 (83.6) 51 (69.9) 22 (30.1) 39 (53.4) 12 (16.4)	1.27 0.004 0.004 0.94 1.27	0.260 0.947 0.947 0.331 0.260	0.64 (0.30–1.93) 0.98 (0.51–1.89) 1.02 (0.53–1.98) 0.74 (0.40–1.36) 1.56 (0.72–3.39)
IL-6 rs1800795 Allele C G Genotype CC CG GG	70 (71.4) 72 (73.5) 26 (26.5) 44 (44.9) 28 (28.6)	42 (57.5) 67 (91.8) 6 (8.2) 36 (49.3) 31 (42.5)	3.57 9.22 9.22 0.33 3.57	0.059 0.002 0.002 0.567 0.059	1.85 (0.98–3.49) 0.25 (0.10–0.64) 4.03 (1.56–10.41) 0.84 (0.46–1.54) 0.54 (0.29–1.03)

χ^2^, chi-square tests were used to determine whether a relationship exists between qualitative data. OR, odds ratio; CI, confidence interval.

**Table 4:** IL-6 rs1800797, IL-6 rs1800795 gene polymorphisms and HPV genotype distribution in cervical cancer patients and controls in the Lithuanian population.

	HPV Type		χ^2^	*p* Value	Number of HPV		χ^2^	*p* Value
	16 + 18 *N* = 72 n (%)	Other *N* = 26 n (%)			1 Type n (%)	>1 Type n (%)		
IL-6 rs1800797 Allele A G Genotype AA AG GG	52 (72.2) 54 (75.0) 18 (25.0) 34 (47.2) 20 (27.8)	23 (88.5) 14 (53.8) 12 (46.2) 11 (42.3) 3 (11.5)	2.81 4.02 4.02 0.19 2.81	0.940 0.045 0.045 0.666 0.094	54 (76.1) 49 (69.0) 22 (31.0) 32 (45.1) 17 (23.9)	21 (77.8) 19 (70.4) 8 (29.6) 13 (48.1) 6 (22.2)	0.03 0.02 0.02 0.08 0.03	0.857 0.896 0.896 0.785 0.857
IL-6 rs1800795 Allele C G Genotype CC CG GG	51 (70.8) 54 (75.0) 18 (25.0) 33 (45.8) 21 (29.2)	19 (73.1) 18 (69.2) 8 (30.8) 11 (42.3) 7 (26.9)	0.05 0.33 0.33 0.10 0.05	0.828 0.568 0.568 0.757 0.828	50 (70.4) 53 (74.6) 18 (25.4) 32 (45.1) 21 (29.6)	20 (74.1) 19 (70.4) 8 (29.6) 12 (44.4) 7 (25.9)	0.13 0.18 0.18 0.003 0.13	0.721 0.668 0.668 0.956 0.721

χ^2^, chi-square tests were used to determine whether a relationship exists between qualitative data.

**Table 5:** IL-6 rs1800797, IL-6 rs1800795 gene polymorphisms and tumor stage in cervical cancer patients.

	Tumor Stage		χ^2^	*p* Value
	I + II *N* = 41 n (%)	III + IV *N* = 41 n (%)		
IL-6 rs1800797 Allele A G Genotype AA AG GG	32 (78.0) 28 (68.3) 13 (31.7) 19 (46.3) 9 (22.0)	28 (68.3) 33 (80.5) 8 (19.5) 20 (48.8) 13 (31.7)	0.99 1.60 1.60 0.05 0.99	0.319 0.206 0.206 0.825 0.319
IL-6 rs1800795 Allele C G Genotype CC CG GG	32 (78.0) 27 (65.9) 14 (34.1) 18 (43.9) 9 (22.0)	28 (68.3) 33 (80.5) 8 (19.5) 20 (48.8) 13 (31.7)	0.99 2.24 2.24 0.20 0.99	0.319 0.135 0.135 0.658 0.319

χ^2^, chi-square tests were used to determine whether a relationship exists between qualitative data.
